# Lung Biopsies in Patients Referred for Allogeneic Hematopoietic Cell Transplantation: Diagnostic Accuracy, Diagnostic Yield, and Clinical Utility

**DOI:** 10.1111/tid.70217

**Published:** 2026-04-16

**Authors:** Sergio Rodriguez‐Rodriguez, Ayman Sayyed, Swe Mar Linn, Caden Chiarello, Mats Remberger, Igor Novitzky‐Basso, Patrik Rogalla, Shahid Husain, Jonas Mattsson

**Affiliations:** ^1^ Messner Bone Marrow Transplant Program Princess Margaret Cancer Centre University Health Network Toronto Ontario Canada; ^2^ Windsor Regional Hospital Schulich School of Medicine & Dentistry Western University Windsor Ontario Canada; ^3^ Department of Medical Sciences (IMV) Clinical Research and Development Unit (KFUE) Uppsala University Hospital Uppsala University Uppsala Sweden; ^4^ Radiology Department Princess Margaret Cancer Centre University Health Network Toronto Ontario Canada; ^5^ Transplant Infectious Diseases Clinic Toronto General Hospital University Health Network Toronto Ontario Canada; ^6^ Gloria and Seymour Epstein Chair in Cell Therapy and Transplantation Department of Medical Oncology and Hematology Princess Margaret Cancer Centre University Health Network (UHN) University of Toronto Toronto Ontario Canada

**Keywords:** allogeneic hematopoietic cell transplantation, fungal infections, graft‐versus‐host disease, lung biopsy

## Abstract

**Background:**

Pulmonary complications after allogeneic hematopoietic cell transplantation (allo‐HCT) are a significant cause of morbidity and mortality. Lung biopsy may help categorize, but its diagnostic yield in this population remains poorly defined.

**Methods:**

We aimed to determine the accuracy (descriptive histopathological report), diagnostic yield (specific histopathologic diagnosis that plausibly explained radiologic abnormalities), complication rates, and clinical utility of lung biopsies performed in patients referred for allo‐HCT at a comprehensive cancer center for pulmonary opacities. We conducted a retrospective cohort study of 76 lung biopsies (image‐guided transthoracic, wedge resection, or transbronchial) performed on 70 patients referred for allo‐HCT.

**Results:**

Lung biopsy was accurate in 93% (*n* = 71/76) of procedures. The diagnostic yield was 72% (*n* = 55/76): malignancy (26%), organizing pneumonia (24%), and infection (16%). Lung biopsy led to a change in management in 51% of procedures (*n* = 39/76); effective (beneficial) in 79% of cases. Major complications occurred in 7% (*n* = 5/76) of procedures: four had a pneumothorax requiring chest tube drainage, and one had an intraprocedural cardiac arrest resulting in death.

**Conclusion:**

In this highly selected cohort of patients referred for allo‐HCT, lung biopsy provided a high diagnostic yield and often altered subsequent management. Integration of biopsy into diagnostic pathways may improve care in selected patients.

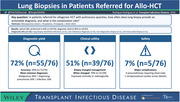

## Graphical abstract

Among patients referred for allogeneic hematopoietic cell transplantation with pulmonary opacities, lung biopsy provided a diagnosis in 72% of procedures and altered management in 51%.

Despite a 7% major complication rate, lung biopsy frequently clarified noninfectious etiologies when bronchoscopy was inconclusive.

**Abbreviations**
Allo‐HCT: allogeneic hematopoietic cell transplantation
ATG: anti‐thymocyte globulin
BAL: bronchoalveolar lavage
cGvHD: chronic graft‐versus‐host disease
CI: confidence interval
CT: computed tomography
DRI: Disease Risk Index
GGO: ground‐glass opacity
GvHD: graft‐versus‐host disease
HCT: hematopoietic cell transplantation
HCT‐CI: hematopoietic cell transplantation‐comorbidity index
HCT‐OP: hematopoietic cell transplantation‐related organizing pneumonia
HL: Hodgkin lymphoma
IQR: interquartile range
KPS: Karnofsky Performance Status
MAC: myeloablative conditioning
NSCLC: non‐small cell lung carcinoma
OP: organizing pneumonia
OS: overall survival
PFT: pulmonary function test
PTCy: post‐transplant cyclophosphamide
RIC: reduced‐intensity conditioning
SE: standard error

## 1 Introduction

Allogeneic hematopoietic cell transplantation (allo‐HCT) is a curative treatment modality for many hematological disorders. Pulmonary complications are common, affecting up to 60% of allo‐HCT recipients, and can be infectious or noninfectious [1, 2]. Earlier autopsy series in adult allo‐HCT recipients showed that noninfectious complications were often only identified postmortem, suggesting substantial under‐recognition [3], with more recent studies continuing to highlight the underdiagnosis of noninfectious entities such as organizing pneumonia (OP) after allo‐HCT [4, 5], one of the most frequent post‐allo‐HCT noninfectious complications [6], associated with an increased risk of non‐relapse mortality [6, 7].

Pre‐allo‐HCT pulmonary function tests (PFTs) are used to identify patients at high‐risk of pulmonary complications and mortality after allo‐HCT [8], while chest computed tomography (CT) detects pulmonary lesions that prompt diagnostic escalation [9]. After allo‐HCT, patients are prone to bacterial and fungal lung infections, often detected on chest CT, followed by bronchoscopy with bronchoalveolar lavage (BAL) to establish the etiologic diagnosis [10, 11], with recent data supporting its use because of its low complication and mortality rates [12]. Yet, BAL may be nondiagnostic or inconclusive, particularly when noninfectious causes are suspected, and lung biopsy may be considered.

Lung biopsies might aid in the diagnosis, but these are often deferred due to substantial risks such as bleeding and pneumothorax. Consequently, most centers use a stepwise strategy that prioritizes bronchoscopy with BAL, with or without transbronchial biopsy, particularly in patients with respiratory failure [13, 14, 15]. Despite this approach, late noninfectious complications remain an unmet need [16], especially in HCT‐related organizing pneumonia (HCT‐OP). The most recent recommendation for patients with HCT‐OP is to reserve lung biopsies for highly selected cases: (1) when the diagnosis remains elusive; (2) when the patient does not show clinical improvement despite corticosteroid treatment; or (3) when the differential diagnosis of malignancy or fungal infection remains high [6, 7].

Therefore, we aimed to assess the diagnostic accuracy, diagnostic yield, and complication profile of lung biopsies for nonspecific focal, multifocal, or diffuse opacities in patients referred for allo‐HCT at a comprehensive cancer center, performed both before and after allo‐HCT.

## 2 Methods

### 2.1 Patients

This observational, retrospective study analyzed lung biopsies performed for nonspecific focal, multifocal, or diffuse opacities in patients referred for allo‐HCT at our institution from January 1, 2010, to June 30, 2022, with detection occurring before and after allo‐HCT. Because such radiologic findings raise similar diagnostic concerns regarding infection, inflammation, or malignancy both before and after allo‐HCT, we included both to analyze the clinical scenarios of all patients assessed within an allo‐HCT program. For brevity, we used the term “lung biopsy” to refer to the three different procedures performed in our center: image‐guided transthoracic biopsies, wedge resections (typically via video‐assisted thoracoscopic surgery), and transbronchial biopsies, with procedure‐specific details provided where relevant. The decision to perform a lung biopsy was at the treating team's discretion. Information regarding Methods is provided in the Supporting Information. All research was conducted in accordance with the Declaration of Helsinki. The Institutional Research Ethics Board approved the study. All patients provided informed consent prior to transplantation, authorizing the use of their clinical data for research purposes.

### 2.2 Biopsy Performance and Complications

Radiologic findings were abstracted from chest radiographs and CT imaging reports, but images were not re‐interpreted for lesion‐level metrics (e.g., target size, attenuation, segmental location, or distance from the pleura). Intraprocedural adverse events were recorded. We report biopsy performance using two metrics: (1) diagnostic accuracy (specimen adequacy), defined as the procurement of tissue sufficient to produce a descriptive histopathology report (i.e., an interpretable pathology report), without requiring repeat invasive diagnostic procedures, and (2) diagnostic yield, defined as the identification of a specific histopathologic diagnosis that plausibly explained the radiologic abnormalities [17, 18]. Biopsies without sufficient tissue were considered inaccurate (either false negative for malignancy or true negative for malignancy). Accurate biopsies with nonspecific or indeterminate findings (e.g., fibrosis or chronic inflammation without a precise diagnosis) were categorized as nondiagnostic. We also evaluated clinical utility, defined as any documented change in the management plan attributed to biopsy results, and clinical benefit, defined as subsequent clinical and/or radiologic improvement following a management change.

### 2.3 Statistical Analysis

Categorical data were presented as counts and proportions, with continuous variables reported as median and interquartile range (IQR); the chi‐square and Mann–Whitney *U* tests were used to compare patient baseline characteristics. Bivariate subgroup comparisons were exploratory; no adjustment for multiple comparisons was performed. All *p*‐values were 2‐sided, considering a *p* < 0.05 as statistically significant. Statistical analyses were performed using Statistica 13 (TIBCO Software Inc., Palo Alto, CA), the open‐source R software (the R Foundation for Statistical Computing, version 4.3.1, Vienna, Austria), and EZR (version 1.68) [19].

## 3 Results

### 3.1 Patient Characteristics

We included 70 patients who underwent 76 lung biopsies: 65 patients had one biopsy, 4 had two biopsies, and 1 had three biopsies. The median age at biopsy was 56 years (IQR 43–64); 45% of the biopsies (*n* = 34/76) were performed on 31 patients before allo‐HCT (median time from biopsy to allo‐HCT was 138 days), and 55% (*n* = 42) in 40 patients after allo‐HCT (median time from allo‐HCT to biopsy was 254 days), with transplant‐related characteristics described in Table 1. Of the patients with two biopsies, two had biopsies both before and after allo‐HCT, while the remaining two had a second biopsy months after the first due to new pulmonary findings. The patient with three biopsies had two of them consecutively, due to the first one being inaccurate, and the third one due to suspicion of relapse of Hodgkin's lymphoma (HL).

**TABLE 1:** Baseline characteristics of patients (*n* = 70) who underwent a lung biopsy.

Characteristics	*n* (%)
Demographic	
Age at biopsy, years, median (IQR)	56 (43–64)
Biological sex	
Male	43 (61)
Female	27 (39)
Transplant‐related	
KPS (*n* = 51)	
≥ 90	42 (82)
< 90	9 (18)
HCT‐CI (*n* = 55)	
≥ 3	28 (51)
< 3	27 (49)
DRI (*n* = 52)	
Low‐risk	4 (8)
Intermediate risk	36 (69)
High‐risk	12 (23)
Conditioning intensity (*n* = 69)	
MAC	23 (33)
RIC	46 (67)
GvHD prophylaxis	
ATG	4 (6)
ATG + PTCy	29 (41)
Others	37 (53)

Abbreviations: ATG, anti‐thymocyte globulin; CT, computed tomography; DRI, Disease Risk Index; HCT‐CI, hematopoietic cell transplantation‐comorbidity index; IQR, interquartile range; KPS, Karnofsky Performance Status; MAC, myeloablative conditioning; PTCy, posttransplant cyclophosphamide; RIC, reduced intensity conditioning.

The radiographic findings on chest CT performed in patients who underwent 73 lung biopsy procedures appeared limited to a single lobe in 52% (*n* = 38/73) and multiple lobes in 48% (*n* = 35/73). The leading radiographic patterns were single or multiple nodules in 37% (*n* = 27/73), GGOs in 26% (*n* = 19), mass or masses in 11% (*n* = 8), consolidation in 10% (*n* = 7), and cavitary lesions in 4% (*n* = 3). Of the remaining nine procedures, eight had two radiographic patterns (three with nodules and GGOs, three with nodules and consolidations, and one each with GGOs and cavitary lesions, and consolidation with a mass lesion), and one had three (nodules, GGOs, and consolidations). Two remaining patients had CT scans prior to their procedure performed outside of our hospital, and these scans were not available for review.

Bronchoscopy with BAL was performed as part of the diagnostic workup before the lung biopsy in 64% (*n* = 45/70) of the patients, with a median interval of 13 days (IQR 6–48) between the bronchoscopy and the lung biopsy. Bronchoscopy had a diagnostic yield of 36% (*n* = 16/45), with specific microbiological results shown in Table 2. In the remaining 64% (*n* = 29), bronchoscopy was nondiagnostic. The diagnostic yield of bronchoscopy did not differ by radiographic pattern. Of the 25 patients without a prior bronchoscopy, the reason for not performing the procedure was rarely documented and appeared to reflect clinician judgment; in two cases, clinical records explicitly noted the absence of bronchoscopy due to the peripheral distribution of the nodules.

**TABLE 2:** Characteristics of patients with a diagnostic bronchoscopy and subsequent lung biopsy.

	CT‐related		Bronchoscopy‐related		Biopsy‐related						Management‐related	
**Patient**	**Lobar involvement**	**Pattern**	**Timing to allo‐HCT**	**Microbiological finding**	**Days since bronchoscopy**	**Reason for biopsy** **^a^**	**Technique**	**Accurate** **^b^**	**Diagnostic** **^b^**	**Result**	**Change**	**Effective**
3	Single	GGOs	After	Influenza B virus CMV	107.00	No improvement	Transbronchial	Yes	Yes	Fibroepithelial polyp	No	—
6	Multiple	Cavitary lesion	Before	Galactomannan (+)	124.00	No improvement	Image‐guided	Yes	No	Inconclusive	No	—
18	Single	Consolidation	After	RSV	1.00	Diagnosis	Image‐guided	No	No	Inaccurate	No	—
30	Single	Cavitary lesion	After	Adenovirus CMV *Propionibacterium acnes*	3.00	Diagnosis	Image‐guided	Yes	Yes	Infectious (*Mucor* spp.)	Yes	Yes
32	Multiple	Consolidation	Before	Galactomannan (+)	30.00	No improvement	Transbronchial	Yes	No	Inconclusive	No	No
33	Multiple	GGOs	After	*Citrobacter freundii* rhinovirus	3.00	Diagnosis	Wedge resection	Yes	Yes	OP^c^	No	No
39	Multiple	Nodule(s)	After	*Aspergillus fumigatus*	0.00	Diagnosis	Transbronchial	Yes	Yes	Malignancy infectious (*Aspergillus* spp.).	Yes	Yes
40	Single	Consolidation	After	MAC Parainfluenza virus	4.00	Diagnosis	Image‐guided	Yes	No	Inconclusive	No	—
43	Multiple	Nodule(s)	Before	*Aspergillus* spp.	80.00	No improvement	Image‐guided	Yes	Yes	Infectious (*Aspergillus* spp.).	Yes	Yes
48	Single	Nodule(s)	Before	*Staphylococcus aureus*	48.00	No improvement	Image‐guided	Yes	No	Inconclusive	No	—
60	Multiple	GGOs	Before	Galactomannan (+)	13.00	Diagnosis	Image‐guided	No	No	Inaccurate	No	—
61	Multiple	Nodule(s)	Before	Influenza A virus	12.00	Diagnosis	Wedge resection	Yes	No	Inconclusive	No	—
62	Multiple	GGOs	After	CMV	13.00	Diagnosis	Wedge resection	Yes	Yes	OP^c^	Yes	Yes
63	Multiple	Nodule(s)	After	*Mycobacterium tuberculosis*	10.00	Diagnosis	Image‐guided	Yes	Yes	Infectious (*Mycobacterium* spp.)	No	—
68	Multiple	GGOs	After	Adenovirus	1.00	Diagnosis	Wedge resection	Yes	Yes	OP^c^	No	—
69	Single	GGOs	Before	Parainfluenza virus *Staphylococcus aureus*	80.00	No improvement	Wedge resection	Yes	Yes	Infectious (*Aspergillus* spp.)	No	—

Abbreviations: allo‐HCT, allogeneic hematopoietic cell transplantation; CMV, cytomegalovirus; CT, computed tomography; GGOs, ground‐glass opacities; MAC, mycobacterium avium complex; OP, organizing pneumonia; RSV, respiratory syncytial virus; spp., species.

^a^ No improvement as the reason for biopsy relates to an identified microorganism explaining the imaging findings, which received appropriate targeted treatment, but was not associated with clinical improvement; or diagnosis as the reason for biopsy involves an identified microorganism that does not account for the imaging findings.

^b^ Diagnostic accuracy was defined as the procurement of tissue sufficient to produce a descriptive histopathology report (i.e., an interpretable pathology report), without requiring repeat invasive diagnostic procedures; diagnostic yield was defined as the identification of a specific histopathologic diagnosis that plausibly explained the radiologic abnormalities.

^c^ Organizing pneumonia (OP) is reported as a histopathologic diagnosis and is not, by itself, a diagnostic of chronic lung graft‐versus‐host disease.

### 3.2 Biopsy Characteristics

Of the 76 performed biopsies, 80% (*n* = 61) were image‐guided, 15% (*n* = 11) were wedge resections, and 5% (*n* = 4) were transbronchial. Overall, 93% (*n* = 71/76) of the procedures were accurate, and the diagnostic yield was 72% (*n* = 55/76): 20 patients had a malignancy, 18 had OP, 12 had infection, and 5 had miscellaneous diagnoses. The remaining 16 accurate biopsies were nondiagnostic, eight due to fibrosis and chronic inflammatory changes, and eight due to no definitive diagnosis or lack of specific pathological findings.

Of the 20 patients with a malignancy reported, 12 had a secondary malignancy, and 8 had a relapse of their hematologic disease. The secondary malignancies diagnosed were non‐small cell lung carcinoma (NSCLC) in eight patients (five with adenocarcinoma, two with squamous cell carcinoma, and one with a poorly differentiated carcinoma), metastatic clear cell renal cell carcinoma, thymoma, posttransplant lymphoproliferative disorder, and a metastatic poorly differentiated squamous cell carcinoma from a primary skin cancer. Relapsed hematologic malignancy was due to acute leukemia in six patients (four myeloid, one lymphoid, and one mixed‐phenotype) and two with HL. Among the 12 patients with proven infection, 7 had a fungal infection, 3 had a mycobacterial infection, 1 had a viral infection, and 1 had nocardiosis; organism‐level microbiological results are detailed in Table 3. Five additional patients had miscellaneous noninfectious diagnoses, comprising fibroepithelial polyp, schwannoma, diffuse alveolar damage, acute fibrinous pneumonia, and extramedullary hematopoiesis.

**TABLE 3:** Microbiological findings of lung biopsy.

	Bacterial	Fungal	Viral	Other
Lung biopsy (*n* = 12)	*Pseudomonas aeruginosa* (2)	*Aspergillus* species (4) *Blastomyces dermatitidis* *Mucor* species *Rhizomucor* species	Epstein‐Barr virus	*Mycobacterium* species (2) *Mycobacterium tuberculosis* *Nocardia* species

*Note*: Some patients had multiple microbiological isolates in the bronchoscopy and bronchoalveolar lavage.

Among the five (7%) remaining patients without a diagnosis (inaccurate biopsy) due to the lack of sufficient tissue for diagnosis, subsequent clinical course confirmed a nonmalignant etiology (true negative) in four cases: three continued treatment for fungal infections (two with improvement, one died due to respiratory failure), and one improved with corticosteroids, whereas one patient underwent a wedge resection and multiple lymph node biopsies, which confirmed diffuse large B‐cell lymphoma (false negative).

Among the 36% of cases (*n* = 16/45) with a positive bronchoscopy result, lung biopsy identified an infectious process in five patients (three confirmations of bronchoscopy findings, two new diagnoses), an alternative noninfectious diagnosis in four (OP in three, malignancy in one), a fibroepithelial polyp in one, nondiagnostic in five, and inaccurate in two. Among the 64% (*n* = 29/45) with a nondiagnostic bronchoscopy, lung biopsy yielded a specific diagnosis in 23 patients: OP in 12, malignancy in 5, infection in 4, and others in 2; the remaining 6 patients had inconclusive results.

### 3.3 Management Changes Due to Biopsy Results

Following biopsy, management (clinical utility) was modified after 39 procedures (51%), remained unchanged in 36 (48%), and was not assessable after 1 procedure (1%) in a patient who had no subsequent follow‐up. Fifteen patients started treatments against a malignancy: eight surgical, three chemotherapy, three radiation, and one chemotherapy with radiation therapy. Antimicrobial therapy was started in 12 patients: antifungal in 7 (3 with a previous diagnostic bronchoscopy), antibacterial in 3, and antituberculosis in 1. One patient received an antifungal with corticosteroids, and one patient received an antifungal and surgical treatment. Nine patients started corticosteroids, eight for OP, one for diffuse alveolar hemorrhage, and one who also received concomitant antifungal therapy. Overall, nine patients underwent surgical intervention, including one in combination with antifungal therapy. Treatment de‐escalation occurred in two patients: one discontinued corticosteroids after starting antibacterial treatment, and one discontinued mycophenolate mofetil.

Among the cohort, 10 patients (13%) who had undergone a previous image‐guided biopsy underwent a wedge resection: 5 for therapeutic and 5 for diagnostic purposes. All 10 patients had an accurate biopsy: 5 had a diagnostic yield, and 5 were nondiagnostic. Among the patients who underwent 39 biopsy procedures and had management modified afterwards, the change resulted in clinical or radiologic improvement (clinical benefit) in 31 procedures (79%), whereas in 8 procedures (21%) no benefit was observed (Figure S1). Two additional patients with nondiagnostic biopsies also had alterations in their management: one following wedge resection and the other based on BAL culture results.

### 3.4 Biopsy Morbidity and Outcomes

At the time of biopsy, the median platelet count was 108 × 10^9^/L (IQR 66–185), and 19 patients (25%) received a platelet transfusion. The median prothrombin time before biopsy was 13.6 s (IQR 12.7–14.9), and the median international normalized ratio was 1.1 (IQR 1.0–1.2).

Complications were observed in 5 (7%) procedures, and 20 (26%) procedures resulted in minor adverse events (Table 4). Four patients developed a large pneumothorax requiring chest tube insertion (two following image‐guided biopsy and two after wedge resection, different from the post‐procedural chest tube). One patient experienced cardiac arrest during the image‐guided biopsy before allo‐HCT due to massive hemoptysis and airway obstruction, required cardiopulmonary resuscitation and endobronchial suctioning, but did not return to spontaneous circulation; of interest, the platelet count was 30 × 10^9^/L, and the patient had received a platelet transfusion before the procedure. Of the 20 patients with minor adverse events requiring intervention, 12 (16%) had a small pneumothorax, 3 (4%) had needle‐track hemorrhage, 2 (3%) had a trace pneumomediastinum (likely from air flow through the needle during the procedure), 1 (1%) had both a needle‐track hemorrhage and small pneumothorax, 1 had a hematoma at the suture site, and 1 patient was unable to tolerate the procedure. The complications occurred after image‐guided biopsies (5%; *n* = 3/61) and wedge resections (19%; *n* = 2/11). A total of 51 (67%) procedures did not result in complications or minor adverse events.

**TABLE 4:** Biopsy‐related complications and outcomes.

Characteristic *n* (%)	Total (*n* = 76)	Pre‐allo‐HCT biopsy (*n* = 34)	Post‐allo‐HCT biopsy (*n* = 42)	*p*
Laboratory, median (IQR)				
Platelet count, × 10^9^/L	108 (66–185)	108 (75–176)	108 (65–187)	0.66
PT, s	13.6 (12.7–14.9)	13.9 (12.8–14.8)	13.1 (12.5–15.1)	0.34
INR	1.1 (1.0–1.2)	1.1 (1.0–1.2)	1.1 (1.0–1.1)	0.23
Platelet transfusion	19 (25)	7 (21)	12 (29)	0.43
Complications	5 (7)	4 (12)	1 (2)	0.23
Minor event	20 (26)	10 (29)	10 (24)	—

Abbreviations: allo‐HCT, allogeneic hematopoietic cell transplantation; INR, International Normalized Ratio; IQR, interquartile range; PT, prothrombin time; S, seconds.

In total, 44 patients died after a median follow‐up of 11.3 months (range 0.3–111.7): 14 due to pulmonary complications, 12 due to relapse of the disease the allo‐HCT was indicated for, 8 due to infections, 6 due to non‐lung GvHD complications, 3 due to miscellaneous reasons, and 1 during the biopsy procedure. Of the 14 patients with pulmonary complications, 8 died due to GvHD‐related complications (6 with clinician‐diagnosed lung GvHD), 3 due to respiratory insufficiency from different causes, and 1 each due to invasive mucormycosis, metastatic renal carcinoma, and complications following double lung transplantation, respectively.

The median OS after lung biopsy was 27.5 months (95% confidence interval [95% CI] 13.7–80.1), with a 1‐year OS of 68.5% (95% CI 56.5–77.8) (Figure S2A). When the analysis was restricted to patients undergoing lung biopsy after allo‐HCT, the 1‐year OS was 50% (95% CI 33.7–64.2) (Figure S2B).

### 3.5 Timing of Biopsy Related to Allo‐HCT

Besides a longer interval time from bronchoscopy to biopsy in patients who underwent biopsy before allo‐HCT (median 41 days) compared to those biopsied after allo‐HCT (median 7 days, *p* = 0.002), there were no significant differences in baseline patient characteristics or in diagnostic accuracy, diagnostic yield, clinical utility, or clinical benefit between patients who had a lung biopsy before and after allo‐HCT (Table S1).

Ten patients had an active malignancy diagnosis before allo‐HCT: six had a hematologic malignancy (four with acute leukemia and two with HL), and four had a solid tumor. Five patients with a hematologic malignancy proceeded to allo‐HCT after chemotherapy treatment; one patient with acute myeloid leukemia underwent allo‐HCT without further treatment, with the myeloid sarcoma resected after transplantation. All six patients experienced relapses after allo‐HCT. Among the four patients with solid tumors, all had NSCLC, comprising three cases of adenocarcinoma and one of squamous cell carcinoma. Two patients (one adenocarcinoma and one squamous cell carcinoma) underwent wedge resection before allo‐HCT and did not develop recurrent NSCLC following transplantation. Of the remaining two patients with adenocarcinoma, one underwent allo‐HCT following right lower lobe lobectomy but subsequently died from relapsed NSCLC, while the second proceeded to allo‐HCT without definitive local therapy. In this latter case, the NSCLC was removed via wedge resection, but the patient ultimately succumbed to relapsed acute myeloid leukemia and lung GvHD.

### 3.6 Timing of Biopsy Related to Chronic Graft‐Versus‐Host Disease

Of the 70 patients included, 44% (*n* = 31/70) developed chronic graft‐versus‐host disease (cGvHD) after allo‐HCT; among these 31 patients, 32% (*n* = 10/31) had a lung biopsy before allo‐HCT, while 68% (*n* = 21/31) had one afterward (1 patient had two). Detailed information on the 21 patients and their 22 biopsies post‐allo‐HCT is shown in Table S2. Of these 22 biopsies, 5 occurred before the diagnosis of cGvHD, and 17 afterward. Among the 17 biopsies performed after the cGvHD diagnosis, 9 were from patients with lung cGvHD involvement; only 2 had a pathological diagnosis of OP, which may be associated with cGvHD but is not universally recognized as a distinctive cGvHD manifestation. Of the nine patients with lung cGvHD, six died: two from lung cGvHD‐related complications, two from non‐lung cGvHD‐related complications, one due to complications following double lung transplantation, and one due to relapse of their hematologic disease.

## 4 Discussion

In this study, we evaluated the diagnostic accuracy, diagnostic yield, and safety profile of lung biopsies in patients being assessed for allo‐HCT who presented with focal, multifocal, or diffuse pulmonary opacities. Among 76 procedures performed, 93% were accurate, and 72% yielded a specific diagnosis, with malignancies, OP, and infections being the most frequent findings. Biopsy results led to modifications in clinical management in 51% of cases (clinical utility), and those changes proved beneficial in 79% of the cases (clinical benefit). Procedure‐related complications occurred in 7% of the procedures, including one fatal event following cardiopulmonary resuscitation. A subset of biopsies was performed on patients who either later developed or already had cGvHD, most often revealing alternative diagnoses like infection or malignancy.

The diagnostic yield of lung biopsies in our cohort was high, but it needs to be interpreted in the context of heterogeneous indications and patient selection. Almost half of the biopsies were performed before allo‐HCT, often to evaluate suspected infection or solid tumors where biopsy is already the standard of care, and the remainder were performed after allo‐HCT in patients with persistent or atypical pulmonary abnormalities. Our findings show that in selected cases where clinicians have already judged that a lung biopsy is appropriate and proceed, the procedure frequently clarifies the diagnosis and affects management; however, biopsies can carry meaningful risk, including rare but deadly situations that might be affected by platelet count. Accurate diagnosis of the underlying cause of pulmonary complications is vital, especially in allo‐HCT patients where invasive fungal pneumonia or mucormycosis is suspected; making quick, accurate decisions is crucial and directly related to the patient's outcome [20, 21].

The interaction between bronchoscopy and lung biopsy merits particular attention. In our series, 64% of patients who underwent lung biopsy had a prior bronchoscopy, and only 36% of those procedures yielded a specific diagnosis. Lung biopsy still provided additional information in a subset of patients, either confirming infection, identifying an alternative noninfectious diagnosis, or prompting a documented change in management, emphasizing the complementary role for lung biopsy after nondiagnostic bronchoscopy in patients where the diagnosis remains elusive, no improvement is found with corticosteroids, or the clinical suspicion of malignancy remains high, consistent with previous studies in immunocompromised patients [22, 23, 24], in which noninvasive methods often yield inconclusive results and invasive diagnostic procedures were required.

Serious procedure‐related complications occurred in 7% of procedures: four patients developed a pneumothorax, requiring chest tube insertion, and only one patient suffered cardiac arrest due to bleeding. Complications occurred after both image‐guided biopsies (5%; *n* = 3/61) and wedge resections (19%; *n* = 2/11); for transbronchial biopsies, the number of procedures was too small to draw safety conclusions or compare them with other series [14]. For image‐guided transthoracic biopsy, the rate of pneumothorax requiring tube thoracostomy was similar to that reported in previous series [24, 25, 26]. The fatal cardiac arrest highlights that, even with careful case selection, lung biopsy still has a small risk of catastrophic complications that could be aggravated by low platelet counts, especially when compared to non‐hematological populations with low mortality rates [27].

Lung biopsy results led to a change in patient management in 51% of cases, most commonly the initiation of chemotherapy or radiotherapy, the commencement or modification of antimicrobial treatment, or the introduction of corticosteroids for OP. Among those with a management change, subsequent clinical or radiologic improvement occurred in 79%, in line with a systematic review and meta‐analysis that reported management changes in 48% of cases across grouped lung biopsy results [12]. Notably, the most clinically impactful diagnoses were noninfectious conditions like malignancy and OP, which often prompted therapeutic changes. These findings support the consideration of integrating lung biopsies into the diagnostic algorithm for allo‐HCT patients with focal pulmonary opacities to improve clinical outcomes. However, we did not observe a clear survival difference according to management change or effectiveness, and the observational design precludes any causal inference about the impact of biopsy on long‐term outcomes.

Timing of the biopsy relative to allo‐HCT did not affect the diagnostic yield or the likelihood of management change, although the clinical questions differed. Pre‐allo‐HCT biopsies were often performed to assess potentially transplant‐limiting malignancy or infection, whereas post‐allo‐HCT biopsies more frequently addressed a differential that included noninfectious complications. We intentionally included biopsies performed at both time points because clinicians encounter similar uncertainty when distinguishing infection, malignancy, or other causes of pulmonary abnormalities. Additionally, evaluating both groups allowed us to assess the safety and clinical utility of lung biopsy throughout the transplant process, during which patients may face procedure‐related risks, such as chemotherapy‐ or disease‐related cytopenias before allo‐HCT, and immunosuppression after allo‐HCT. This highlights the real‐world experience that a transplant program encounters during pretransplant evaluation and post‐allo‐HCT care. It is important to note a significant difference in the number of days from bronchoscopy to biopsy: 41 days before allo‐HCT versus 7 days after allo‐HCT. This difference may result from varying follow‐up practices and the risk of pulmonary complications before and after allo‐HCT.

Our study has several limitations. First, its retrospective design and modest sample size limit the ability to establish causal relationships and may introduce unmeasured confounding and selection bias. Second, we included only patients referred for allo‐HCT who had a lung biopsy before or after transplantation, and we did not systematically identify patients with similar radiologic findings who were managed without biopsy. As a result, we cannot compare outcomes between patients who did and did not undergo a lung biopsy, and the selected patients may represent a group with more severe, atypical, or persistent abnormalities. Moreover, because this was a transplant program‐focused retrospective study, we did not reanalyze CT images and could not evaluate lesion‐level radiographic predictors. Similarly, granular bronchoscopy technique variables were not consistently reported and were not analyzed. Finally, the cohort is heterogeneous with respect to timing, radiographic patterns, and underlying diagnoses, and a substantial proportion of management changes were driven by malignancy treatment, for which biopsy is already a standard of care. Although the single‐center design limits generalizability, it ensures consistency in biopsy techniques, periprocedural care, and patient management, thereby strengthening internal validity.

In this single‐center cohort of patients referred for allo‐HCT who underwent lung biopsies before or after transplant, lung biopsies often yielded a specific diagnosis that informed subsequent management. However, complications occurred, including a procedure‐related death, emphasizing that these are high‐stakes elective diagnostic procedures. Our results support a selective, multidisciplinary risk‐benefit approach to lung biopsy in this population rather than routine use and highlight the need for prospective multicenter studies to better define patient selection and optimal diagnostic pathways.

## Author Contributions

S.R.R., A.S., and J.M. designed the research study. S.R.R., A.S., S.M.L., and C.C. gathered the information. S.R.R., A.S., and M.R. performed the statistical analysis. S.R.R. and A.S. drafted the initial manuscript version. All authors contributed to the interpretation of results, provided critical revisions, and approved the final manuscript.

## Funding

The authors have nothing to report.

## Ethics Statement

The Research Ethics Board at the University Health Network, Toronto, approved the study (REB #22‐5744).

## Conflicts of Interest

The authors declare no conflicts of interest.

## Supporting information




**Supporting File 1**: tid70217‐sup‐0001‐SuppMat.docx.

**Supporting File 2**: tid70217‐sup‐0002‐VisualAbstract.jpg.

## Data Availability

The data are available upon reasonable request to the corresponding author and subject to institutional approval.
