# Supplementary material for: Lung Biopsies in Patients Referred for Allogeneic Hematopoietic Cell Transplantation: Diagnostic Accuracy, Diagnostic Yield, and Clinical Utility
Source: Transpl Infect Dis. 2026 Apr 16;28(3):e70217. doi: 10.1111/tid.70217 (PMC13262550; doi:10.1111/tid.70217)
Supplement: Supplementary file 1 — Supporting File 1: tid70217‐sup‐0001‐SuppMat.docx. [file TID-28-e70217-s001.docx]

**SUPPLEMENTARY MATERIAL**

**MATERIALS AND METHODS**

**Patients**

For allo-HCT recipients, we analyzed the age at the time of allo-HCT, the performance status (Karnofsky Performance Status or KPS), the Hematopoietic Cell Transplantation-Comorbidity Index (HCT-CI) score, the Disease Risk Index, donor type (matched sibling, matched unrelated, or partially mismatched donors), graft manipulation (T cell depletion), the stem cell source (bone marrow or mobilized peripheral blood stem cells), the presence and severity of acute graft-versus-host disease (aGvHD) and chronic graft-versus-host disease (cGvHD), and the use of immunosuppressive therapy (such as cyclosporine and methylprednisolone) at the time of lung biopsy.

Additionally, we determined the history of pre-allo-HCT pulmonary complications, the type of lung biopsy procedure, the timing related to allo-HCT, the respiratory symptoms or imaging findings that prompted the procedure, the neutrophil engraftment status, concurrent infectious illness (bacterial, viral, or fungal infection or colonization), and antimicrobial, antifungal, or antiviral therapy at the time of biopsy, including prophylactic and treatment agents. We documented the treatment changes after the lung biopsy, the start and discontinuation of medications, as well as surgical and malignancy treatments. We captured the patient's survival status and, if pertinent, the cause of death; all observations were censored at the date of death or at the last follow-up for living patients. When multiple biopsies were performed in a single patient, follow-up was censored at the time of the following biopsy.

**Conditioning regimens and graft-versus-host-disease prophylaxis**

The conditioning regimens were classified as reduced intensity (RIC) or myeloablative (MAC). RIC regimens included fludarabine 35 mg/m^2^ for four days starting from day -5, busulfan 3.2 mg/kg for two days starting from day -5, and total body irradiation (TBI) at a dose of 2 Gy on day -1; fludarabine 35 mg/m^2^ daily for four days and treosulfan 10 g/m^2^ or 14 g/m^2^ daily for three days.

MAC regimens included any of the following: fludarabine 35 mg/m^2^ for four days starting from day -5, busulfan 3.2 mg/kg for four days starting from day -5, with or without TBI at a dose of 4 Gy on day -1; cyclophosphamide 60 mg/kg for two days starting on day -5 and TBI at a dose of 2 Gy twice daily for three days starting on day -3; fludarabine 40 mg/m^2^ for three days starting from day -5 and TBI at a dose 2 Gy twice daily for three days starting on day -3; etoposide 60 mg/kg once on day -5 and TBI at a dose of 2 Gy twice daily for three days starting on day -3.

GvHD prophylaxis regimens included any of the following: rabbit anti-thymocyte globulin (ATG) 2 mg/kg or 4.5 mg/kg, post-transplant cyclophosphamide (PTCy) 50 mg/kg for two days starting on day +3 and cyclosporine A (CsA) 2.5 mg/kg every 12 hours starting on day +5; PTCy 50 mg/kg for two days starting on day +3, mycophenolate mofetil (MMF) 15 mg/kg every 8 hours starting on day +5, and CsA 2.5 mg/kg every 12 hours starting on day +5; ATG 2 mg/kg, methotrexate (MTX) 15 mg/m^2^ on day +1 and 10 mg/m^2^ on days +3 and +6, and CsA 2.5 mg/kg every 12 hours starting on day -1; CsA 2.5 mg/kg every 12 hours starting on day -1 and MMF 15 mg/kg every 12 hours starting on day +1; alemtuzumab 60 mg subcutaneously on day -3 and CsA 2.5 mg/kg every 12 hours starting on day +5; or CsA 2.5 mg/kg every 12 hours starting on day -1 and MTX 15 mg/m^2^ on day +1 and 10 mg/m^2^ on days +3 and +6.

**Biopsy techniques**

The study utilized three biopsy techniques at different times: image-guided transthoracic biopsies, wedge resections, and transbronchial biopsies, all performed at the Toronto General Hospital. Image-guided transthoracic biopsies, either by CT or ultrasound (US), began with a preliminary US or CT scan of the chest to target the lesion for biopsy. The US was used to navigate the needle into targets with pleural contact, and CT-fluoroscopy was used for all other target lesions. A core biopsy was performed using either a 20-Gauge core needle with a tray or an 18-Gauge biopsy gun, based on the target size, depth, access route, and the operator’s preference. All biopsies concluded with a post-procedure CT scan to rule out complications. Bronchoscopy procedures could include bronchial washing and BAL, as well as transbronchial lung biopsies and brushing. We did not consider BAL-only procedures as lung biopsies. Wedge resections were performed with or without prior bronchoscopy with BAL, typically via video-assisted thoracoscopic surgery (VATS) to locate the lesion, followed by a wedge resection.

**Survival outcomes**

Overall survival (OS) was calculated from both the biopsy and allo-HCT dates, until death or last follow-up, with survivors censored at last follow-up. The cumulative incidence of relapse (CIR) was estimated from the date of transplant to relapse with non-relapse mortality (NRM) as the competing event. NRM was calculated from the date of transplant to death from any cause other than relapse. The Kaplan-Meier method was used to estimate OS, with comparisons performed using the log-rank test. CIR and NRM were analyzed using Fine and Gray's competing-risks model.

**SUPPLEMENTARY TABLES**

**Table S1. Biopsy-related characteristics and their comparison to allogeneic hematopoietic cell transplantation timing.**

| **Characteristic**  **n (%)** | **Total**  **(n=76)** | **Pre-allo-HCT biopsy**  **(n=34)** | **Post-allo-HCT biopsy**  **(n=42)** | **p** |
| --- | --- | --- | --- | --- |
| **Diagnostic-related** |  |  |  |  |
| **Radiographical findings (n=73)** |  |  |  |  |
| Lobar | 38 (52) | 20 (59) | 18 (43) | 0.22 |
| Multilobar | 35 (48) | 13 (38) | 22 (52) |  |
| **Bronchoscopy** |  |  |  |  |
| Days from bronchoscopy to biopsy, median (IQR) | 13 (6–48) | 41 (16–80) | 7 (4–18) | **0.002** |
| Diagnostic | 16 (21) | 7 (21) | 9 (21) | 0.33 |
| **Biopsy** |  |  |  |  |
| Successful | 71 (93) | 31 (91) | 40 (95) | 0.65 |
| Diagnostic | 55 (72) | 23 (68) | 32 (76) | 0.45 |
| Infectious | 12 (16) | 5 (15) | 7 (16) |  |
| Malignancy | 20 (26) | 10 (29) | 10 (24) |  |
| Organizing pneumonia | 18 (24) | 6 (18) | 12 (29) |  |
| Others | 5 (6) | 2 (6) | 3 (7) |  |
| **Management plan** |  |  |  |  |
| Plan changed | 39 (52) | 17 (50) | 22 (54) | 0.82 |
| Beneficial change | 31/39 (79) | 14/17 (82) | 17/22 (77) | NS |
| Treatment added |  |  |  |  |
| Antineoplastic | 15 (20) | 8 (23) | 7 (17) | NS |
| Infectious | 12 (16) | 7 (21) | 5 (12) | NS |
| Corticosteroid | 9 (12) | 1 (3) | 8 (19) | NS |
| Surgical | 9 (12) | 6 (18) | 3 (7) | NS |
| Wedge resection | 10 (13) | 7 (21) | 3 (7) | 0.10 |
| Radiological improvement | 51 (67) | 27 (79) | 24 (57) | 0.16 |

**Abbreviations:** allo-HCT: allogeneic hematopoietic cell transplantation; CT: computed tomography; IQR: interquartile range.

**Legend: *** Diagnostic accuracy was defined as the procurement of tissue sufficient to produce a descriptive histopathology report (i.e., an interpretable pathology report), without requiring repeat invasive diagnostic procedures; diagnostic yield was defined as the identification of a specific histopathologic diagnosis that plausibly explained the radiologic abnormalities. Clinical utility was defined as any documented change in the management plan attributed to biopsy results; clinical benefit was defined as subsequent clinical and/or radiologic improvement following a management change.

**Table S2. Characteristics of patients with a lung biopsy post-allogeneic hematopoietic cell transplantation with chronic graft-versus-host disease.**

|  | **cGvHD-related** | | | | **CT-related** | | **Bronchoscopy-related** | | **Biopsy-related** | | | | |
| --- | --- | --- | --- | --- | --- | --- | --- | --- | --- | --- | --- | --- | --- |
| **Patient** | **Timing**  **of biopsy** | **Severity** | **Lung**  **cGvHD** | **Days to**  **cGvHD** | **Lobar**  **involvement** | **Pattern** | **Performed** | **Result** | **Technique** | **Accurate†** | **Diagnostic†** | **Result** | **Complication** |
| 1 | After Dx | Severe | Yes | 688 | Multiple | GGOs | Yes | No abnormality | Wedge resection | Yes | Yes | OP | No |
| 2 | After Dx | Mild | No | 489 | NR | NR | No | - | Image-guided | Yes | Yes | Malignancy | No |
| 3 | After Dx | Severe | No | 439 | Single | GGOs | Yes | Influenza B,  CMV | Transbronchial | Yes | Yes | Other | No |
| 4 | After Dx | Severe | Yes | 114 | Multiple | GGOs | Yes | No abnormality | Wedge resection | Yes | Yes | Infectious | No |
| 8 | Before Dx | Severe | Yes | -17 | Multiple | Nodule(s),  consolidation | Yes | No abnormality | Image-guided | Yes | No | Inconclusive | Small PTX |
| 13 | Before Dx | Moderate | Yes | -19 | Single | Nodule(s) | No | - | Image-guided | Yes | Yes | OP | No |
| 18 | Before Dx | Severe | Yes | -125 | Single | Consolidation | Yes | RSV | Image-guided | No | No | - | No |
| 26 | After Dx | Moderate | Yes | 915 | Multiple | Nodule(s) | No | - | Image-guided | Yes | Yes | Malignancy | Small PTX |
| 28 | After Dx | Moderate | No | 407 | NR | NR | No | - | Image-guided | Yes | Yes | Infectious | No |
| 31 | After Dx | Severe | No | 1099 | Single | Consolidation | Yes | No abnormality | Image-guided | Yes | Yes | Other | Small PTX |
| 36 | After Dx | Severe | Yes | 22 | Single | GGOs | Yes | No abnormality | Wedge resection | Yes | Yes | OP | No |
| 37 | After Dx | Severe | No | 453 | Single | Mass(es) | No | - | Image-guided | Yes | Yes | Malignancy | Small PTX |
| 39 | After Dx | Severe | Yes | 37 | Multiple | Nodule(s) | Yes | *A. fumigatus* | Transbronchial | Yes | Yes | Malignancy | No |
| 40 | After Dx | Moderate | Yes | 1001 | Single | Consolidation | Yes | MAC,  Parainfluenza | Image-guided | Yes | No | Inconclusive | No |
| 41 | After Dx | Severe | Yes | 716 | Multiple | GGOs | Yes | No abnormality | Image-guided | Yes | No | Inconclusive | Small PTX |
| 49 | After Dx | Moderate | No | 1116 | Single | Nodule(s) | No | - | Image-guided | Yes | Yes | Malignancy | No |
| 53 | Before Dx | Moderate | Yes | -157 | Multiple | Nodule(s) | No | - | Image-guided | Yes | Yes | Infectious | No |
| 58 | After Dx | Severe | Yes | 1372 | Single | Nodule(s) | No | - | Image-guided | Yes | No | Inconclusive | No |
| 58 | After Dx | Severe | No | 1542 | Single | Nodule(s) | No | - | Image-guided | Yes | No | Inconclusive | Small PTX |
| 62 | Before Dx | Moderate | Yes | 0 | Multiple | GGOs | Yes | CMV | Wedge resection | Yes | Yes | OP | No |
| 66 | After Dx | Moderate | No | 80 | Multiple | GGOs | Yes | No abnormality | Wedge resection | Yes | Yes | OP | No |
| 67 | After Dx | Severe | Yes | 1156 | Multiple | Nodule(s),  GGOs | Yes | No abnormality | Transbronchial | Yes | No | Inconclusive | No |

**Abbreviations:** cGvHD: chronic graft-versus-host disease; CMV: cytomegalovirus; CT: computed tomography; Dx: cGvHD diagnosis; GGOs: ground-glass opacities; MAC: Mycobacterium avium complex; NR: not reported; OP: organizing pneumonia; PTX: pneumothorax; RSV: respiratory syncytial virus.

**Legend:** * Lung chronic graft-versus-host disease (cGvHD) was defined as a new-onset of an obstructive lung defect indicative of bronchiolitis obliterans syndrome. **†** Diagnostic accuracy was defined as the procurement of tissue sufficient to produce a descriptive histopathology report (i.e., an interpretable pathology report), without requiring repeat invasive diagnostic procedures; diagnostic yield was defined as the identification of a specific histopathologic diagnosis that plausibly explained the radiologic abnormalities. ‡ Organizing pneumonia (OP) is reported as a histopathologic diagnosis and is not, by itself, a diagnostic of lung chronic graft-versus-host disease.

**FIGURE LEGENDS**

**Figure S1. Sankey plot for type of biopsy, diagnostic procedure, change in management, and management efficacy.**

**Figure S2. One-year overall survival (OS) after lung biopsy (A) and after allogeneic hematopoietic cell transplantation (B).**
