# Supplementary figures and images for: Lung Biopsies in Patients Referred for Allogeneic Hematopoietic Cell Transplantation: Diagnostic Accuracy, Diagnostic Yield, and Clinical Utility
Source: Transpl Infect Dis. 2026 Apr 16;28(3):e70217. doi: 10.1111/tid.70217 (PMC13262550; doi:10.1111/tid.70217)

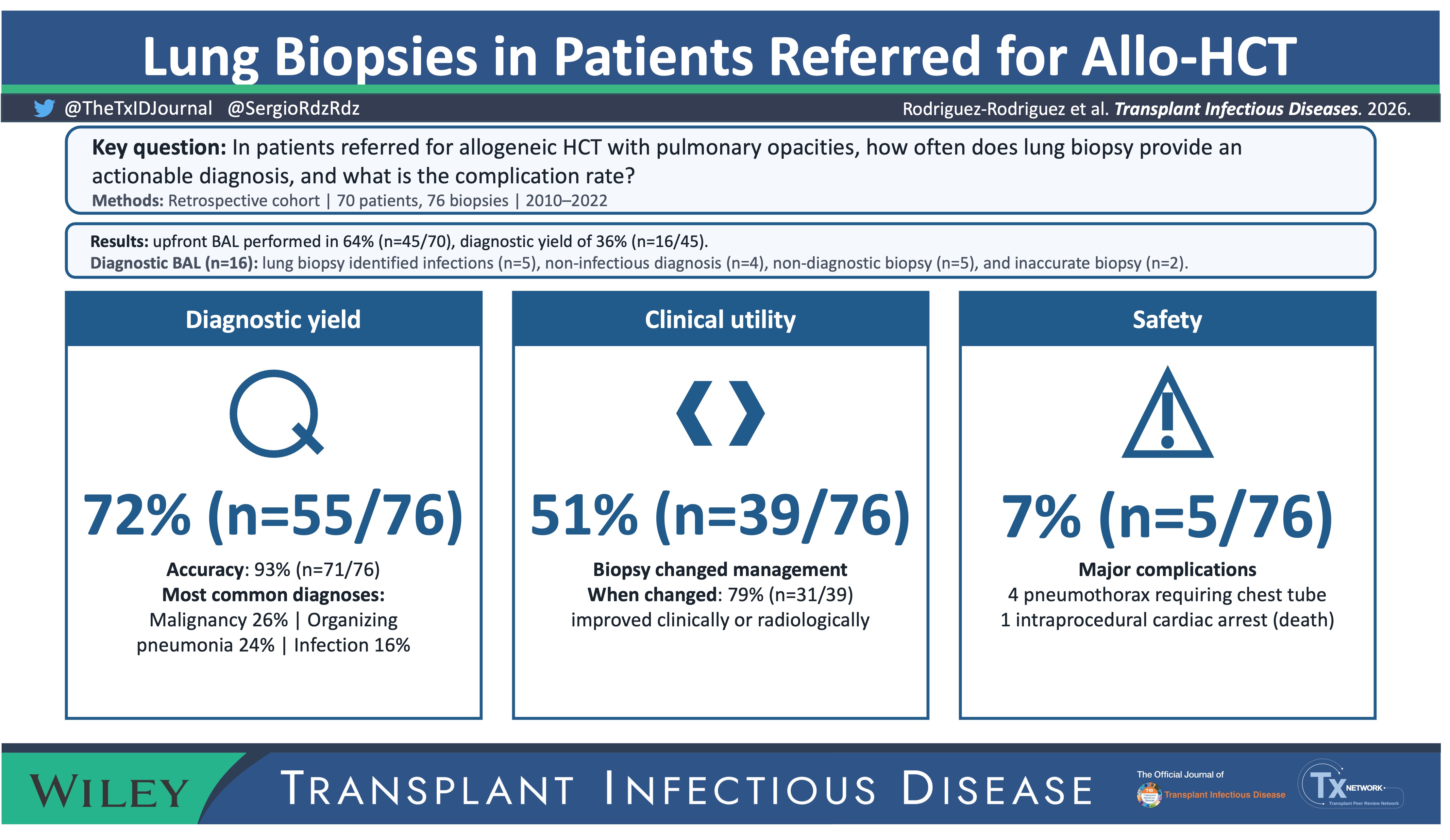

Supplement: Supplementary file 2 — Supporting File 2: tid70217‐sup‐0002‐VisualAbstract.jpg. [file TID-28-e70217-s002.jpg]
